# An *Adcy3* coding mutation causes partial loss of enzymatic function, contributing to obesity in a rat model by reducing lipolysis

**DOI:** 10.21203/rs.3.rs-8309561/v1

**Published:** 2026-01-09

**Authors:** Mackenzie K. Fitzpatrick, Osborne Seshie, Christina Scott, Anusha Vora, Mary E. Seramur, Angela Beeson, Leighelle Adrian, Michael Grzybowski, Jason Klotz, Aron M. Geurts, Chia-Chi Chuang Key, Leah C. Solberg Woods

**Affiliations:** 1 Department of Internal Medicine, Section on Molecular Medicine, Wake Forest University School of Medicine, Winston-Salem NC, USA; 2 Department of Physiology, Medical College of Wisconsin, Milwaukee WI, USA; 3 Department of Internal Medicine, Section on Pulmonary, Critical Care, Allergy and Immunologic Diseases, Wake Forest University School of Medicine, Winston-Salem NC, USA

**Keywords:** obesity, *Adcy3*, lipolysis, thermogenesis, rat model

## Abstract

We previously showed that rats with a protein-coding mutation in *Adenylate cyclase 3 (Adcy3)* (Adcy3^mut/mut^) have increased adiposity. ADCY3 catalyzes the production of cyclic AMP (cAMP), a key secondary messenger that regulates lipolysis and thermogenesis. Here, we assessed how Adcy3^mut/mut^ affects lipolysis, thermogenesis, and cAMP signaling. Adcy3^mut/mut^ and wild-type (WT) males and female rats were fed a high-fat diet for 12 weeks. We measured body weight, fat mass, serum free fatty acids (FFA) during a 48-hour fast, body temperature during acute cold exposure, and triglyceride lipase gene expression after a 48-hour fast and after prolonged cold exposure. We also measured cAMP production in response to a β−3 adrenergic receptor agonist (CL 316,243) in adipose tissue *ex vivo*. Adcy3^mut/mut^ rats displayed increased adiposity, decreased serum FFA, and downregulated adipose triglyceride lipase gene expression. Additionally, cAMP production was decreased in Adcy3^mut/mut^ adipose tissue compared with WT adipose tissue in response to *ex vivo* stimulation with CL 316,243. Adcy3^mut/mut^ females, but not males, showed a trend toward decreased body temperature during acute cold exposure. These findings demonstrate that a mutation in the transmembrane domain of ADCY3 results in partial loss of enzymatic function, decreasing lipolytic responsiveness and contributing to increased adiposity.

## 2 Introduction

Obesity rates, as well as rates of associated comorbidities, have been steadily increasing since the early 1990s with levels of overweight and obesity in US adults projected to reach 80% by 2050.[1–3] Several factors, including genetics and the environment, contribute to a person’s risk of developing obesity.[4] Specific signaling pathways, including thermogenesis and lipolysis in adipose tissue, have been identified as key regulators of body weight and composition.[5–7]

The gene *Adenylate cyclase 3 (Adcy3)* has been repeatedly associated with obesity in both humans and rodent models.[8] The ADCY3 protein localizes to the plasma membrane with upstream G-protein coupled receptors in white (WAT) and brown (BAT) adipose tissue.[9–11] In adipose tissue, fasting or cold exposure releases catecholamines that activate the β−3 adrenergic receptor (β3-AR), releasing G-proteins that bind to ADCY3 and drive the conversion of ATP to cyclic AMP (cAMP).[12, 13] Then, cAMP regulates lipolysis and thermogenesis primarily through activation of protein kinase A (PKA), which phosphorylates key downstream targets including hormone sensitive lipase (HSL) and adipose triglyceride lipase (ATGL).[10, 11, 13–17] These targets then promote triglyceride hydrolysis, upregulate uncoupling protein 1 (UCP1) to stimulate heat production, and activate transcription factors such as peroxisome proliferator-activated receptor gamma (PPARG) and CCAAT/enhancer-binding protein alpha (CEBPα) to influence adipogenesis.[10, 11, 13–17]

In humans, both protein-coding and expression-altering variants in *ADCY3* have been associated with monogenic and polygenic obesity.[18–23] Multiple rodent studies have reported that *Adcy3* knockout (KO) mice have increased adiposity, increased food intake, decreased cAMP production, and decreased lipolysis and thermogenesis, although most of these studies have been done only in males.[16, 24–26] Studies have also shown that mice with an *Adcy3* gain of function mutation exhibit increased energy expenditure (EE).[27] We previously identified[28] and validated [29, 30] a protein-coding mutation in the second transmembrane (TM) helix of ADCY3 (Adcy3^mut/mut^) that is associated with obesity in a rat model. Mutations in this same TM region have been associated with height adjusted body mass index in humans[18], demonstrating translational relevance.

We previously found that while Adcy3^mut/mut^ rats had increased adiposity relative to wild-type (WT) rats, the cause of adiposity differed by sex: male Adcy3^mut/mut^ rats showed increased food intake, while female Adcy3^mut/mut^ rats showed decreased EE.[30] Similar results were found in a rat model of a partial *Adcy3* KO (Adcy3^+/−^)[29], supporting an important role of *Adcy3* in adiposity. Given the known role of cAMP signaling in lipolysis and thermogenesis, coupled with recent literature showing impaired thermogenesis in *Adcy3* KO mice[16, 31] and the sex-specific decreased EE in our initial study[30], we set out to understand how Adcy3^mut/mut^ affects these signaling pathways in adipose tissue. We performed fasting and cold exposure experiments to investigate lipolysis and thermogenesis, and we assessed molecular signaling downstream of ADCY3 to understand the mechanistic changes driving these phenotypes. Studies were conducted in both Adcy3^mut/mut^ and Adcy3^+/−^ to both verify previous mouse KO studies and to compare the *in vivo* effects of a protein-coding mutation vs. diminished *Adcy3* expression. In addition to the *in vivo* experiments in this study, we also performed an *ex vivo* study to assess the impact of the Adcy3^mut/mut^ on adipose tissue cAMP production in response to stimulation with a β3-AR agonist. Importantly, although obesity-associated protein coding variants in *Adcy3* have been identified in humans[8], with the exception of our previous work [30], no rodent models of protein-coding mutations in *Adcy3* have been studied.

## 3 Methods

### 3.1 Animals and Diet

Adcy3^mut/mut^ and Adcy3^+/−^ rats were generated using CRISPR-SpCas9 and obtained from colleagues at the Medical College of Wisconsin as described previously.[30] Both Adcy3^+/−^ and Adcy3^mut/mut^ were generated on the Wistar Kyoto background. Heterozygous breeding pairs were used to establish colonies at the Wake Forest University School of Medicine. WT and Adcy3^+/−^ or Adcy3^mut/mut^ rats were saved for experiments. Rats were weaned at 3 weeks of age and pair-housed with another rat of the same sex and genotype. Rats were housed at standard temperature and humidity on a 12-hour light/dark cycle with *ad libitum* access to food and water. All animal experiments were performed using protocols approved by the Institutional Animal Care and Use Committee at Wake Forest and in compliance with ARRIVE guidelines. All methods were performed in accordance with the relevant institutional guidelines and regulations.

Heterozygous Adcy3^+/−^ rats have ~50% expression of *Adcy3*[30] and were used as “KO” experimental animals due to homozygous lethality in *Adcy3* KO rats. Adcy3^mut/mut^ rats, which do not display this lethality, have a protein coding mutation (F122delV123L) in the second ADCY3 TM helix.[30]

8 rats per genotype-sex group (Adcy3^mut/mut^ and Adcy3^+/−^ with respective WT for each group) were collected for thermogenesis and lipolysis experiments. A separate, second group of rats (7–12 per group, Adcy3^mut/mut^ with respective WT only) was collected for a 24-hour fast before necropsy. A third group of rats (6 per group, Adcy3^mut/mut^ and Adcy3^+/−^ with respective WT) was used to collect tissues for real-time quantitative PCR (rt-qPCR). A fourth group of rats (6 per group, Adcy3^mut/mut^ with respective WT only) was used to collect adipose tissue to assess cAMP production in response to a β3-AR agonist *ex vivo*.

All rats were fed standard rodent chow (Lab Diet, St. Louis MO, Prolab RMH 3000, #5P00) until 5 weeks of age. To maintain experimental consistency with our prior work[30], all rats used for fasting and cold exposure experiments, 24-hour fast, and tissue collection for rt-qPCR began a high-fat diet (HFD) (60% kcal fat, 20% kcal protein, 20% kcal carbohydrates, ResearchDiet, New Brunswick NJ, D12492) at 5 weeks of age and continued HFD until necropsy. Rats used for the *ex vivo* study were placed on either HFD or standard chow for 9 weeks prior to tissue collection.

### 3.2 Group 1: Thermogenesis and Lipolysis, Study Design

Beginning at HFD start/0 weeks on diet (WOD), body weight was measured weekly. Fat mass and lean mass were measured in triplicate via EchoMRI (EchoMRI LLC, Houston TX) at 7 WOD (Figure 1a). Between 10 and 12 WOD, rats received 3 tests: a 48-hour fast, an acute cold exposure, and a prolonged cold exposure (Figure 1a).

#### 3.2a 48-Hour Fast and Serum FFA

Rats were fasted for 48 hours beginning at 9 AM, and serum was collected via tail snip at 0, 24, and 48 hours. Serum FFA was measured with a Serum/Plasma Fatty Acid Detection Kit (ZenBio, Durham NC, #SFA-5).

#### 3.2b Acute Cold Exposure

Body temperature was measured to assess cold-induced thermogenesis as described previously.[31] Rats were individually housed in a cold room maintained at 4°C for 5 hours during the light cycle. Rats had access to water, but not food, during the cold exposure, and cages were free of bedding and other insulating materials. Body temperature was measured with a rectal thermometer every 15 minutes for the first hour, then every 30 minutes. The temperature probe was cleaned with 70% ethanol between rats. Animal welfare was also assessed at each time point.

#### 3.2c Prolonged Cold Exposure before Necropsy

Rats underwent a 48-hour cold exposure immediately before necropsy to induce expression of lipolysis and thermogenesis genes as described previously.[32] Rats were individually housed and retained *ad libitum* access to food and water. Rats were continuously housed in a cold room maintained at 4°C with the same light cycle and humidity conditions as standard housing. Animal welfare checks were performed every hour for the first 6 hours, then once every 12 hours. For necropsy, rats were brought from the cold room to the necropsy space one-by-one with the cage placed on ice packs to minimize time spent at room temperature before euthanasia. Necropsy tissues were used to measure expression of triglyceride lipase genes and thermogenic genes.

#### 3.2d Necropsy

After a 4-hour fast at 12 WOD, rats were euthanized via live decapitation using a rat guillotine. The chest cavity was opened and diaphragm punctured with scissors. There was no need to use any chemical agents. The following tissues were weighed and snap-frozen in liquid nitrogen: liver, retroperitoneal visceral white fat pads (RetroFat), gonadal fat, BAT, interscapular subcutaneous adipose (SubQ). We also dissected, weighed and snap-froze the brain. Kidneys, omental fat (OmenFat), pancreas, and heart were also weighed but not snap-frozen.

### 3.3 Group 2: 24-Hour Fast before Necropsy

A separate, second group of rats was fasted for 24 hours before necropsy at 7 WOD to analyze expression of triglyceride lipase genes under these conditions. Rats retained *ad libitum* access to water. Aside from the differences in age and fasting before necropsy, procedures were the same as described in Section 3.2d.

### 3.4 Group 3: RT-qPCR of Adipogenic Genes

RetroFat was collected from a third group of rats not exposed to prolonged cold or fasting. RT-qPCR experiments were performed as described previously.[30] Briefly, RNA was extracted from RetroFat, SubQ, and BAT and converted to complementary DNA (cDNA) with a High-Capacity cDNA Reverse Transcription Kit (Applied Biosystems, Waltham MA, 4368814), and RT-qPCR was run with PowerUp SYBR Green Master Mix (Applied Biosystems, A25741) with *β-actin* (Integrated DNA Technologies, Coralville IA) as a reference gene. Fold change was calculated using the 2^−ΔΔCt^ method.[33] Sequences for all primers (Integrated DNA Technologies) are shown in **Table S1**. Necropsy procedures were the same as described above in Section 3.2d.

### 3.5 Group 4: *Ex Vivo* Assessment of Adipose Tissue cAMP

Rats were euthanized via live decapitation without fasting. Per a previously published protocol[34], with modifications made to measure cAMP, RetroFat was rapidly dissected and placed in Dulbecco’s phosphate buffered saline (DPBS) (Gibco). RetroFat was then cut into 50 mg (+/− 10 mg) sections, and sections were placed into a 24-well plate with DPBS. After all wells were filled, DPBS was removed and replaced with media containing Dulbecco’s Modified Eagle Medium (DMEM) without phenol red (Gibco), 5% bovine serum albumin (BSA) (Fisher Bioreagents) and 500 μM 3-isobutyl-1-methylxanthine (IBMX) (Sigma Aldrich). Sections were incubated at 37°C for 10 minutes. Media was then removed and replaced with either control (DMEM/BSA/IBMX as described above) or treated (DMEM/BSA/IBMX plus 3 μM CL 316,243 (C5976 Sigma-Aldritch)) media. Samples were then incubated at 37°C for 0, 5, or 20 minutes, after which media was removed and tissue was placed in lysis buffer (0.1 M HCl + 0.1% Triton + 500 μM IBMX) and homogenized using a bead beater with zirconium beads (Biospec Products) at 2000 RPM. Homogenate was centrifuged for 10 minutes at 16,000 xg, and supernatant was stored at −80°C. cAMP content was measured using cAMP ELISA Assay (ENZO ADI-900–066A) per manufacturer’s instructions.

### 3.6 Statistical Analysis

Data were analyzed using either RStudio (2023.09.1) or GraphPad Prism (10.2.2). Outliers were identified by Grubb’s Test or by the ROUT method in Prism with Q=1%. Data were assessed for normality with the Shapiro-Wilk test and, if not normal, Mann-Whitney U test was used in place of a Student’s t-test. Sphericity was assessed with Mauchly’s Test for repeated measures ANOVA (rmANOVA). Greenhouse-Geisser correction was applied as needed.

Comparisons were made between WT and Adcy3^+/−^ or Adcy3^mut/mut^ rats within each strain, not between strains, due to differences in WT phenotypes between colonies. Within each strain, data were first analyzed using a two-way ANOVA or rmANOVA with genotype and sex as factors. Given the large differences in body weight and fat mass between sexes, all data were then stratified by sex and analyzed using an unpaired Student’s t-test or a one-way rmANOVA between genotypes. Triglyceride lipase gene expression was first analyzed using a three-way ANOVA with sex, genotype, and lipase gene as factors to assess broad differences in gene expression when examining both genes together. The data were then stratified to assess specific differences within each sex and gene. Due to large sex differences in adiposity phenotypes, we only report the outcomes of the stratified analyses. cAMP data was stratified by sex and analyzed using a two-way rmANOVA with genotype and diet as factors.

## 4 Results

### 4.1 Adcy3^mut/mut^ rats have increased HFD-induced adiposity and less serum FFA during a prolonged fast relative to WT rats.

Supporting our previous work[30], Adcy3^mut/mut^ males (p=0.0002) and Adcy3^mut/mut^ females (p=0.0008) gained more weight than WT rats over the course of the study (**Figure S1a**). After 7 WOD, Adcy3^mut/mut^ males (p<0.0001) and females (p<0.0001) had more fat mass in EchoMRI than WT rats without any significant differences in lean mass (Figure 1b). Similarly, at necropsy, Adcy3^mut/mut^ males and females had significantly more RetroFat (p<0.0001, p<0.0001), gonadal fat (p<0.0001, p=0.0046), and OmenFat (p=0.0001, p=0.0181) than WT rats (**Figure S1b**). Adcy3^mut/mut^ males had significantly larger pancreases (p=0.0383) at necropsy than WT males, but there were no differences in the necropsy weights of any other organs (**Figure S1c**).

We hypothesized that the observed increase in adiposity could result from impaired lipolysis and thermogenesis, two key catabolic pathways in adipose tissue that are mediated by cAMP signaling. To test our hypothesis, we performed a 48-hour fast to unmask lipolytic defects that may be challenging to detect under fed conditions and measured serum FFA as a readout of lipolysis. In Adcy3^mut/mut^, there was a significant genotype effect on serum FFA over 48 hours (p=0.0230), where Adcy3^mut/mut^ rats had significantly lower serum FFA relative to WT rats (Figure 1c). There were no significant sex or interaction effects in the two-way rmANOVA. These data show that Adcy3^mut/mut^ rats have increased fat accumulation, likely due to impaired lipolysis.

### 4.2 Adcy3^mut/mut^ females, but not males, may have impaired thermoregulation during acute cold exposure.

We next examined another catabolic pathway, thermogenesis, by performing cold exposure studies. In the overall analysis, there was a significant sex effect for acute cold body temperature in Adcy3^mut/mut^ (p<0.0001), where females had a higher body temperature relative to males. When stratified by sex, there were no genotypic differences in body temperature in Adcy3^mut/mut^ males relative to WT males during the cold exposure (Figure 2). However, Adcy3^mut/mut^ females (p=0.0620) tended to maintain a lower body temperature during the cold exposure than WT females (Figure 2). This decreased body temperature suggests that Adcy3^mut/mut^ females, but not males, may have impaired cold-induced thermogenesis.

### 4.3 Adcy3^mut/mut^ rats have decreased expression of lipase genes.

We sought to further understand the molecular basis of these phenotypes, so we examined the expression of two key genes that regulate the first and second steps of lipolysis: *Patatin-like phospholipase domain containing 2* (*Pnpla2)* and *Lipase E* (*Lipe)*, corresponding to ATGL and HSL, respectively. We assessed these genes both in tissues collected after prolonged cold exposure and after a 24-hour fast.

In tissues collected after the prolonged cold exposure, in a three-way ANOVA with sex, genotype, and gene as factors, there was a significant genotype effect in Adcy3^mut/mut^ for RetroFat (p<0.0001) and SubQ (p=0.0023) with no effect in BAT (Figure 3a). These results demonstrate that lipase genes are broadly downregulated in visceral and subcutaneous WAT in Adcy3^mut/mut^ rats relative to WT rats.

The data were then stratified, and differences between genotypes within each sex and gene were assessed. In Adcy3^mut/mut^ males, there were decreases in *Lipe* in RetroFat (p=0.0334) and in *Pnpla2* in RetroFat (p=0.0021) and SubQ (p=0.0218) (Figure 3a). In Adcy3^mut/mut^ females, there was a trend toward decreased *Pnpla2* in RetroFat (p=0.0855) (Figure 3a). These data support the conclusion that lipolysis is impaired in Adcy3mut/mut.

Furthermore, after a 24-hour fast: in the three-way ANOVA, there were significant sex (p=0.0150) and sex by genotype interaction (p=0.0105) effects in RetroFat, as well as significant sex (p=0.0132) and genotype (p=0.0212) effects in SubQ, again demonstrating broad differences between genotypes in these lipolysis genes in WAT (Figure 3b). In the stratified analysis, *Pnpla2* in RetroFat was lower in Adcy3^mut/mut^ males relative to WT males (p=0.0228) (Figure 3b). In Adcy3^mut/mut^ females, there were decreases in *Lipe* in SubQ (p=0.0244) and in *Pnpla2* in both SubQ (p=0.0008) and BAT (p=0.0494) (Figure 3b).

Overall, these data demonstrate that lipolysis genes are downregulated in Adcy3^mut/mut^ in multiple tissues across two different lipolysis-inducing experimental conditions (prolonged cold and fasting).

### 4.4 Adcy3^mut/mut^ do not have altered expression of Ucp1 or adipogenesis genes.

Next, we investigated if genes involved in thermogenesis and adipogenesis were altered in adipose tissue, given that these pathways are closely linked to lipolysis and energy balance. We measured expression of *Ucp1*, a mitochondrial protein that plays a critical role in thermogenesis, in both BAT and SubQ WAT in tissues from necropsy following prolonged cold exposure. There were no significant differences in *Ucp1* expression between genotypes in either tissue, although there was very large variation in *Ucp1* expression, particularly in WAT (Figure 4a).

We also measured expression of *Pparg* and *Cebpa*, two master transcription factors that play a central role in adipogenesis, in RetroFat under baseline ad libitum conditions (e.g., not exposed to prolonged cold or fasting). There were no significant changes in *Pparg* or in *Cebpa* in either sex compared with WT (Figure 4b). These data indicate that thermogenic and adipogenic gene expression is unaltered in Adcy3^mut/mut^.

### 4.5 Adipose tissue from Adcy3^mut/mut^ rats has decreased *ex vivo* cAMP production in response to stimulation with a β3-AR agonist.

In *ex vivo* adipose tissue stimulated with CL 316,243 (a β3-AR agonist), after stratifying by sex, both Adcy3^mut/mut^ males (p=0.0094) and Adcy3^mut/mut^ females (p=0.0046) had significantly lower cAMP production over time relative to WT rats (Figure 5). There was a significant effect of diet in males (p=0.042) where the cAMP signal is blunted on HFD (**Figure S2a**), with no diet effect in females (**Figure S2b**). These findings demonstrate that Adcy3^mut/mut^ causes a partial loss of enzymatic function that may drive the decreased lipolysis and increased adiposity observed in Adcy3^mut/mut^ rats.

### 4.6 Similar results were observed in Adcy3^+/−^ rats.

Broadly, experimental results in Adcy3^+/−^ were similar to results observed in Adcy3^mut/mut^. Adcy3^+/−^ males and females gained more weight, although this was only significant in females (p=0.0730, p=0.0270, **Figures S3a**), had more fat mass in EchoMRI (p=0.0006, p=0.0004) without any differences in lean mass (**Figure S3b**), and had significantly more RetroFat (p<0.0001, p=0.0019), gonadal fat (p=0.0002, p=0.0013), and OmenFat (p=0.0004, p=0.0017) at necropsy than WT rats (**Figure S3c**). Adcy3^+/−^ males also had significantly larger pancreases (p=0.0186) at necropsy than WT males with no differences in the necropsy weights of any other organs (**Figure S3d**).

When measuring serum FFA after a 48-hour fast, there was a significant genotype by time interaction effect (p=0.0140) where at 48 hours, both Adcy3^+/−^ males (p=0.0182) and females (p=0.0963) had lower serum FFA than WT rats (**Figure S3e**). We also found a significant effect of sex on serum FFA over 48 hours (p=0.0001), where females had higher FFA relative to males. Adcy3^+/−^ females (p=0.0005), but not males, maintained a significantly lower body temperature during acute cold exposure than WT rats (**Figure S3f**), indicating impaired cold-induced thermogenesis and in agreement with what we observed in Adcy3^mut/mut^ rats.

When we measured lipase gene expression after cold exposure in Adcy3^+/−^, there was a significant genotype effect in Adcy3^+/−^ for both RetroFat (p=0.0024) and SubQ (p<0.0001) with no effect in BAT, aligning with results in Adcy3^mut/mut^ (**Figure S4a**). When the data were stratified, in Adcy3^+/−^ males, there were decreases in *Lipe* in SubQ (p=0.0612) and in *Pnpla2* in RetroFat (p=0.0130), SubQ (p=0.0582), and BAT (p=0.0248) (**Figure S4a**). In Adcy3^+/−^ females, there were decreases in *Lipe* in RetroFat (p=0.0003) and SubQ (p=0.0344) and in *Pnpla2* in SubQ (p=0.0214) (**Figure S4a**). 24-hour fast tissues were only available for the Adcy3^mut/mut^ strain. These data support the conclusion that lipolysis is also impaired in Adcy3^+/−^. There were no genotypic differences in *Ucp1* expression in BAT or SubQ in Adcy3^+/−^ (**Figure S4b**). Similarly, there were no genotypic differences in *Cebpa* in RetroFat in Adcy3^+/−^(**Figure S4c**), although there was a significant decrease in *Pparg* in Adcy3^+/−^ females relative to WT females (p=0.0377; **Figure S4c**). Overall, these findings in Adcy3^+/−^ parallel our findings in Adcy3^mut/mut^, providing additional support that a decrease in *Adcy3* function leads to reduced lipolytic responsiveness to prolonged fasting and cold exposure, thereby contributing to increased adiposity.

## 5 Discussion

In the present study, we demonstrate that a TM mutation in *Adcy3* causes a partial loss of enzymatic function where Adcy3^mut/mut^ rats have decreased *ex vivo* cAMP production leading to decreased lipolytic responsiveness and contributing to increased body weight and fat mass compared with WT rats. We also saw that Adcy3^mut/mut^ females, but not males, had a trend toward decreased body temperature during acute cold exposure. Collectively, these data demonstrate the importance of a TM coding variant in *Adcy3* and show that *Adcy3* plays a critical role in regulating adipose tissue metabolism, including lipolysis in both sexes and thermogenesis in females.

During fasting, lipolysis generates FFAs as an alternative energy source for the body, breaking down triglycerides stored in WAT to do so, resulting in smaller WAT depots.[15] cAMP can also drive lipolysis by activating various downstream effectors that regulate lipolysis and thermogenesis.[10, 13–17] We hypothesized that the observed increase in adiposity could result from impaired lipolysis and thermogenesis, two key catabolic pathways in adipose tissue that are mediated by cAMP signaling. In our study, during a 48-hour fast, Adcy3^mut/mut^ rats of both sexes had decreased serum FFA levels, suggesting decreased lipolysis.[15, 35] Adcy3^mut/mut^ males and females also showed decreased expression of triglyceride lipase genes in WAT under both prolonged cold exposure and 24-hour fast conditions, further supporting the conclusion that lipolysis is impaired. Similar results are seen in Adcy3^+/−^ rats (**Figures S3 and S4**), providing additional evidence of *Adcy3’s* role in lipolytic responsiveness to fasting or prolonged cold. Although previous studies in Adcy3^+/−^ mice have reported that FFA levels were unchanged relative to WT mice in response to β-AR stimulation[25], a separate group found that HSL phosphorylation in WAT was lower in Adcy3^+/−^ relative to WT mice, supporting the idea that *Adcy3* impairment decreases lipolysis.[16] Collectively, the decreases we observed in both lipase gene expression and serum FFAs support the hypothesis that impaired lipolysis in Adcy3^mut/mut^ rats contributes to increased adiposity.

These findings are further supported by *ex vivo* data where we show that adipose tissue cAMP production in response to stimulation with a β3-AR agonist is significantly lower in both male and female Adcy3^mut/mut^ rats relative to WT rats. This decrease in cAMP production is particularly important, demonstrating that this TM mutation leads to a partial loss of enzymatic function that may underlie the decreased lipolytic responsiveness and ultimately increased adiposity in Adcy3^mut/mut^ rats. Although previous studies observed decreased cAMP in *Adcy3* KO mice using *Adcy3* enzymatic assays *in vitro* with tissue homogenates[16, 25], ours is the first study to examine cAMP production in response to a β3-AR agonist using intact *ex vivo* tissues from an *Adcy3*-deficient genetic model. These results reveal the importance of the ADCY3 TM domain for protein function and for regulation of lipolysis.

Although decreased cAMP production and lipolytic responsiveness were evident in both male and female Adcy3^mut/mut^ rats, we observed genotypic differences in cold-induced thermoregulation only in females. Specifically, Adcy3^mut/mut^ females maintained slightly lower body temperatures and Adcy3^+/−^ females exhibited significantly lower body temperatures than WT females, indicating impaired thermogenesis. In contrast, no differences in body temperature were noted between Adcy3^+/−^ or Adcy3^mut/mut^ males relative to WT males. These findings align with our initial study of Adcy3^mut/mut^, where we reported that Adcy3^mut/mut^ (and Adcy3^+/−^) females, but not males, had decreased EE.[30] Importantly, our previous work found decreased EE in Adcy3^mut/mut^ (and Adcy3^+/−^) females when rats were not fasted and were at room temperature, demonstrating that these genotypic differences in thermoregulation in females are consistent across conditions. Previous studies support the existence of sex differences in thermogenesis, potentially explaining our findings.[36–40] However, prior to the present study, thermogenesis had not been directly measured during cold exposure in female *Adcy3* KO mice, although impaired cold-induced thermogenesis has previously been reported in male mice that have decreased ADCY3 expression in BAT due to knock-down of *receptor expression-enhancing protein 6* (*Reep6*).[31] Given that there are well-established differences in thermogenesis between mice and rats[41], previous findings may not be directly comparable to the present study. Nevertheless, our data indicate a potential sex-dependent role for ADCY3 in regulating cold-induced thermoregulation and EE in rats. Unfortunately, studies examining how sex hormones may affect *Adcy3* expression, or the expression of other *Adcy* isoforms, are lacking. Future studies that investigate the influence of sex hormones in WAT in this model will be key to understanding these sex differences.

Given these findings, we might have also expected to see decreased *Ucp1* in Adcy3^mut/mut^ females, especially since a previous study of Adcy3^+/−^ mice reported decreased *Ucp1* in epididymal WAT after an overnight fast (no cold exposure).[16] However, given that we observed unusually high *Ucp1* expression in these cold-exposed tissues (RT-qPCR, CT value < 17 in BAT and < 24 in SubQ), it is possible that our 48-hour cold exposure before necropsy excessively upregulated *Ucp1* in all groups, masking any differences between genotypes. Future studies are needed to test this hypothesis.

Finally, we did not observe any consistent differences between genotypes in the expression or activation of *Pparg* or *Cebpa*. These markers promote adipogenesis in WAT[42], so the lack of differences in these markers suggests that adipogenesis is likely not impacted by Adcy3^mut/mut^ (or by Adcy3^+/−^). A previous study did report increased *Pparg* and *Cebpa* in Adcy3^+/−^ mice after an overnight fast.[16] Given the stablished differences in adipose tissue metabolism between mice and rats[41], it is possible that these disparities in fasting conditions, species, and strain explain the differences in our results here. Furthermore, previous studies have established that the interplay of lipid anabolism and catabolism can be regulated by hormonal cues, insulin, and fasting/fed status, raising the possibility that variables not measured here are influencing these markers[43]. Future studies of Adcy3^mut/mut^ in an adipocyte cell culture model will provide more precise insight into whether altered adipogenesis can contribute to adiposity specifically in Adcy3^mut/mut^.

In conclusion, our findings show that a loss-of-function mutation in *Adcy3* leads to impaired lipolytic responsiveness to fasting and cold exposure in WAT, contributing to the adiposity phenotype observed in Adcy3^mut/mut^ rats. We also show that Adcy3^mut/mut^ (and Adcy3^+/−^) females, but not males, have impaired thermogenesis, supporting our earlier work showing that Adcy3^+/−^ and Adcy3^mut/mut^ females have decreased EE under non-fasted, room-temperature conditions.[30] These data show that a protein-coding mutation in the TM domain of ADCY3 can influence adipose tissue metabolism, ultimately increasing adiposity.

## Supplementary Material

Supplementary Files

This is a list of supplementary files associated with this preprint. Click to download.

- FitzpatrickSupplementCombined1282025.pdf

## Figures and Tables

**Figure 1.** Study design, adiposity, and serum FFA in Adcy3^mut/mut^ vs WT rats **(a)** Timeline of the study shown in weeks on diet (WOD). Rats received an EchoMRI before experiments to measure lipolytic response and thermogenesis: 48-hour fast, acute cold exposure, and prolonged cold exposure before necropsy (sac) after 12 WOD. **(b)** Adcy3^mut/mut^ males (M) and females (F) have more fat mass in EchoMRI after 7 WOD than wild-type (WT) rats without differences in lean mass. **(c)** Adcy3^mut/mut^ M and F have less serum FFA than WT rats over 48 hours, as shown by a significant main genotype effect in the two-way ANOVA. HFD: high-fat diet. Mean ± SEM. T-test or rmANOVA, *p<0.05, ****p<0.0001

**Figure 2.** Body temperature during acute cold exposure in Adcy3^mut/mut^ vs WT rats. During an acute 5-hour cold exposure at 4°C, there were no significant differences in body temperature between Adcy3^mut/mut^ males (M) and wild-type (WT) M. Adcy3^mut/mut^ females (F) tend to maintain a lower body temperature during acute cold exposure than WT F. Mean ± SEM. rmANOVA.

**Figure 3.** Triglyceride lipase gene expression in retroperitoneal fat (RetroFat), subcutaneous white adipose tissue (SubQ), and brown adipose tissue (BAT) Adcy3^mut/mut^ vs WT rats. **(a)** After a prolonged 48-hour cold exposure at 4°C, Adcy3^mut/mut^ males (M) and females (F) generally have lower lipase gene expression (*Lipase E (Lipe)* and *Patatin-like phospholipase domain containing 2* (*Pnpla2))* in RetroFat and SubQ than wild-type (WT) rats, as shown by both three-way ANOVA (results on legend) and stratified analyses (results in graph). No genotypic differences in general lipase gene expression in BAT after prolonged cold exposure. **(b)** Adcy3^mut/mut^ M and F also have lower lipase gene expression in RetroFat and SubQ after a 24-hour fast. No genotypic differences in general lipase gene expression in BAT after a 24-hour fast, except that Adcy3^mut/mut^ F express less *Pnpla2* than WT rats. HSL: Hormone-sensitive lipase, ATGL: Adipose triglyceride lipase. Mean ± SEM. T-test or three-way ANOVA, *p<0.05, **p<0.01, ***p<0.001, ****p<0.0001

**Figure 4.** *Ucp1* and adipogenic gene expression in adipose tissue in Adcy3^mut/mut^ vs WT rats. **(a)** After a prolonged 48-hour cold exposure at 4°C, there were no significant differences in *Uncoupling protein 1* (*Ucp1)* expression in Adcy3^mut/mut^ males (M) or females (F) in brown adipose tissue (BAT) or subcutaneous adipose (SubQ) relative to WT rats. **(b)** After standard necropsy (no fasting or cold exposure), there were no significant differences in *Peroxisome proliferator-activated receptor gamma* (*Pparg)* or *CCAAT/enhancer-binding protein alpha* (*Cebpa)* expression in RetroFat between genotypes. Mean ± SEM. T-test.

**Figure 5.** *Ex vivo* cAMO production in response to stimulation with a β3-AR agonist in RetroFat of Adcy3^mut/mut^ vs WT rats. Retroperitoneal fat pads (RetroFat) from Adcy3^mut/mut^ males (M) and Adcy3^mut/mut^ females (F) have less cAMP production *ex vivo* in response to stimulation with a β3-AR agonist than wild-type (WT) rats over 20 minutes, as shown by a significant main effect of genotype in a two-way rmANOVA. Data shown here are combined across diets. Mean ± SEM. rmANOVA, **p<0.01

## Data Availability

The datasets generated and/or analyzed during the current study are available from the corresponding author on reasonable request.

## References

[1] World Obesity Atlas. 2023; Available from: https://data.worldobesity.org/publications/?cat=19.

[2] Duis J. and Butler M.G., Syndromic and Nonsyndromic Obesity: Underlying Genetic Causes in Humans. Adv Biol (Weing), 2022. 6(10): p. e2101154. 10.1002/adbi.202101154 35680611

[3] National-level and state-level prevalence of overweight and obesity among children, adolescents, and adults in the USA, 1990–2021, and forecasts up to 2050. Lancet, 2024. 404(10469): p. 2278–2298. 39551059 10.1016/S0140-6736(24)01548-4 PMC11694015

[4] Apovian V.M., Obesity: definition, comorbidities, causes, and burden. Am J Manag Vare, 2016. 22(7 Suppl): p. s176–85. 27356115

[5] Della Guardia L. and Sgin A.V., Obesity-induced tissue alterations resist weight loss: A mechanistic review. Diabetes Obes Metab, 2024. 26(8): p. 3045–3057. 38720199 10.1111/dom.15637

[6] London E. and Stratakis V.A., The regulation of PKA signaling in obesity and in the maintenance of metabolic health. Pgarmacol Tger, 2022. 237: p. 108113. 10.1016/j.pharmthera.2022.108113 35051439

[7] Wen X., , Signaling pathways in obesity: mechanisms and therapeutic interventions. Signal Transduct Target Tger, 2022. 7(1): p. 298. 10.1038/s41392-022-01149-x PMC9420733 36031641

[8] Fitzpatrick M. and Solberg Woods L.V., Adenylate cyclase 3: a potential genetic link between obesity and major depressive disorder. Pgysiol Genomics, 2024. 56(1): p. 1–8. 10.1152/physiolgenomics.00056.2023 PMC11281808 37955134

[9] Defer N., Best-Belpomme M., and Hanoune J., Tissue specificity and physiological relevance of various isoforms of adenylyl cyclase. Am J Pgysiol Renal Pgysiol, 2000. 279(3): p. F400–16. 10.1152/ajprenal.2000.279.3.F400 10966920

[10] Vgaudgry A., , Perinatal expression of adenylyl cyclase subtypes in rat brown adipose tissue. Am J Pgysiol, 1996. 270(4 Pt 2): p. R755–60. 10.1152/ajpregu.1996.270.4.R755 8967404

[11] Devasani K. and Yao Y., Expression and functions of adenylyl cyclases in the CNS. Fluids Barriers VNS, 2022. 19(1): p. 23. 10.1186/s12987-022-00322-2 PMC8935726 35307032

[12] Vero V., , β3-Adrenergic receptors regulate human brown/beige adipocyte lipolysis and thermogenesis. JVI Insiggt, 2021. 6(11). 10.1172/jci.insight.139160 PMC8262278 34100382

[13] London E., Bloyd M., and Stratakis V.A., PKA functions in metabolism and resistance to obesity: lessons from mouse and human studies. J Endocrinol, 2020. 246(3): p. R51–R64. 32485681 10.1530/JOE-20-0035 PMC7385994

[14] Ding L., , Reduced lipolysis response to adipose afferent reflex involved in impaired activation of adrenoceptor-cAMP-PKA-hormone sensitive lipase pathway in obesity. Sci Rep, 2016. 6: p. 34374. 27694818 10.1038/srep34374 PMC5046068

[15] Madsen L. and Kristiansen K., The importance of dietary modulation of cAMP and insulin signaling in adipose tissue and the development of obesity. Annals of tge New York Academy of Sciences, 2010. 1190(1): p. 1–14. 10.1111/j.1749-6632.2009.05262.x 20388132

[16] Tong T., , Adenylyl cyclase 3 haploinsufficiency confers susceptibility to diet-induced obesity and insulin resistance in mice. Sci Rep, 2016. 6: p. 34179. 27678003 10.1038/srep34179 PMC5039768

[17] Vao W., , p38 mitogen-activated protein kinase is the central regulator of cyclic AMP-dependent transcription of the brown fat uncoupling protein 1 gene. Mol Vell Biol, 2004. 24(7): p. 3057–67. 10.1128/MCB.24.7.3057-3067.2004 PMC371122 15024092

[18] Stergiakouli E., , Genome-wide association study of height-adjusted BMI in childhood identifies functional variant in ADCY3. Obesity (Silver Spring), 2014. 22(10): p. 2252–9. 25044758 10.1002/oby.20840 PMC4265207

[19] Voisin S., , Many obesity-associated SNPs strongly associate with DNA methylation changes at proximal promoters and enhancers. Genome Med, 2015. 7: p. 103. 26449484 10.1186/s13073-015-0225-4 PMC4599317

[20] Saeed S., , Loss-of-function mutations in ADCY3 cause monogenic severe obesity. Nat Genet, 2018. 50(2): p. 175–179. 29311637 10.1038/s41588-017-0023-6

[21] Grarup N., , Loss-of-function variants in ADCY3 increase risk of obesity and type 2 diabetes. Nat Genet, 2018. 50(2): p. 172–174. 29311636 10.1038/s41588-017-0022-7 PMC5828106

[22] Loid P., , Rare Variants in Genes Linked to Appetite Control and Hypothalamic Development in Early-Onset Severe Obesity. Front Endocrinol (Lausanne), 2020. 11: p. 81. 32153512 10.3389/fendo.2020.00081 PMC7047210

[23] Toumba M., , Molecular modelling of novel ADCY3 variant predicts a molecular target for tackling obesity. Int J Mol Med, 2022. 49(1). 10.3892/ijmm.2021.5065 PMC8651229 34821371

[24] Siljee J.E., , Subcellular localization of MC4R with ADCY3 at neuronal primary cilia underlies a common pathway for genetic predisposition to obesity. Nat Genet, 2018. 50(2): p. 180–185. 29311635 10.1038/s41588-017-0020-9 PMC5805646

[25] Wang Z., , Adult type 3 adenylyl cyclase-deficient mice are obese. PLoS One, 2009. 4(9): p. e6979. 19750222 10.1371/journal.pone.0006979 PMC2735775

[26] Vao H., , Disruption of type 3 adenylyl cyclase expression in the hypothalamus leads to obesity. Integr Obes Diabetes, 2016. 2(2): p. 225–228. 27942392 10.15761/IOD.1000149 PMC5145199

[27] Pitman J.L., , A gain-of-function mutation in adenylate cyclase 3 protects mice from diet-induced obesity. PLoS One, 2014. 9(10): p. e110226. 25329148 10.1371/journal.pone.0110226 PMC4199629

[28] Keele G.R., , Genetic Fine-Mapping and Identification of Candidate Genes and Variants for Adiposity Traits in Outbred Rats. Obesity (Silver Spring), 2018. 26(1): p. 213–222. 29193816 10.1002/oby.22075 PMC5740008

[29] Fitzpatrick M.K., , A Mutation in the Transmembrane Domain of Adenylate Cyclase 3 Impairs Enzymatic Function to Cause Sex-Specific Depression- and Anxiety-Like Behaviors and Food Seeking in a Rat Model. Genes Brain Begav, 2025. 24(3): p. e70028. 10.1111/gbb.70028 PMC12149762 40492272

[30] Fitzpatrick M.K., , Protein-coding mutation in Adcy3 increases adiposity and alters emotional behaviors sex-dependently in rats. Obesity (Silver Spring), 2024. 10.1002/oby.24178 PMC12247540 39632398

[31] Son Y., , REEP6 knockout leads to defective β-adrenergic signaling in adipocytes and promotes obesity-related metabolic dysfunction. Metabolism, 2022. 130: p. 155159. 35150731 10.1016/j.metabol.2022.155159

[32] Guezennec V.Y., , Hormonal and metabolic response to physical exercise, fasting and cold exposure in the rat. Effects on ketogenesis in isolated hepatocytes. Eur J Appl Pgysiol Occup Pgysiol, 1988. 57(1): p. 114–9. 10.1007/BF00691249 3277845

[33] Ballester M., , Real-time quantitative PCR-based system for determining transgene copy number in transgenic animals. Biotecgniques, 2004. 37(4): p. 610–3. 10.2144/04374ST06 15517974

[34] Bridge-Vomer P.E. and Reilly S.M., Measuring the Rate of Lipolysis in Ex vivo Murine Adipose Tissue and Primary Preadipocytes Differentiated In Vitro. J Vis Exp, 2023(193). 10.3791/65106 PMC10583296 37010285

[35] Rydén M., , Lipolysis defect in people with obesity who undergo metabolic surgery. J Intern Med, 2022. 292(4): p. 667–678. 35670497 10.1111/joim.13527 PMC9540545

[36] Gómez-García I., , Sexual Dimorphism in Brown Adipose Tissue Activation and White Adipose Tissue Browning. Int J Mol Sci, 2022. 23(15). 10.3390/ijms23158250 PMC9368277 35897816

[37] Löfgren P., , Major Gender Differences in the Lipolytic Capacity of Abdominal Subcutaneous Fat Cells in Obesity Observed before and after Long-Term Weight Reduction. Tge Journal of Vlinical Endocrinology & Metabolism, 2002. 87(2): p. 764–771. 10.1210/jcem.87.2.8254 11836318

[38] Rodriguez-Vuenca S., , Sex-dependent thermogenesis, differences in mitochondrial morphology and function, and adrenergic response in brown adipose tissue. J Biol Vgem, 2002. 277(45): p. 42958–63. 10.1074/jbc.M207229200 12215449

[39] Ostrom K.F., , Physiological roles of mammalian transmembrane adenylyl cyclase isoforms. Pgysiol Rev, 2022. 102(2): p. 815–857. 10.1152/physrev.00013.2021 PMC8759965 34698552

[40] Warrington N.M., Sun T., and Rubin J.B., Targeting brain tumor cAMP: the case for sex-specific therapeutics. Front Pgarmacol, 2015. 6: p. 153. 10.3389/fphar.2015.00153 PMC4516881 26283963

[41] Hankenson F.V., , Effects of Rodent Thermoregulation on Animal Models in the Research Environment. Vomp Med, 2018. 68(6): p. 425–438. 10.30802/AALAS-CM-18-000049 PMC6310197 30458902

[42] Habinowski S.A. and Witters L.A., The effects of AICAR on adipocyte differentiation of 3T3-L1 cells. Biocgem Biopgys Res Vommun, 2001. 286(5): p. 852–6. 10.1006/bbrc.2001.5484 11527376

[43] Zgang D., , Important Hormones Regulating Lipid Metabolism. Molecules, 2022. 27(20). 10.3390/molecules27207052 PMC9607181 36296646

